# Deep Image Steganography Using Transformer and Recursive Permutation

**DOI:** 10.3390/e24070878

**Published:** 2022-06-26

**Authors:** Zhiyi Wang, Mingcheng Zhou, Boji Liu, Taiyong Li

**Affiliations:** School of Economic Information Engineering, Southwestern University of Finance and Economics, Chengdu 611130, China; wangzy_t@swufe.edu.cn (Z.W.); 220081203008@smail.swufe.edu.cn (M.Z.); 220081202004@smail.swufe.edu.cn (B.L.)

**Keywords:** image steganography, data hiding, deep learning, transformer, image encryption

## Abstract

Image steganography, which usually hides a small image (hidden image or secret image) in a large image (carrier) so that the crackers cannot feel the existence of the hidden image in the carrier, has become a hot topic in the community of image security. Recent deep-learning techniques have promoted image steganography to a new stage. To improve the performance of steganography, this paper proposes a novel scheme that uses the Transformer for feature extraction in steganography. In addition, an image encryption algorithm using recursive permutation is proposed to further enhance the security of secret images. We conduct extensive experiments to demonstrate the effectiveness of the proposed scheme. We reveal that the Transformer is superior to the compared state-of-the-art deep-learning models in feature extraction for steganography. In addition, the proposed image encryption algorithm has good attributes for image security, which further enhances the performance of the proposed scheme of steganography.

## 1. Introduction

With the development of computer technology and communication technology, a large number of images are stored in the cloud and transmitted and shared via the internet. How to keep some sensitive images, such as military images, medical images or personal privacy images from being accessed by unauthorized persons, has become an important branch of information security [1,2,3]. One direct way is to encrypt the images by changing the positions and values of the pixels in images so that there does not exist any visually meaningful information in the images. The chaos-based image encryption models emerging in recent years are such methods. Despite their great success in privacy protection, they also suffer from the clear disadvantage that crackers can see at a glance that the images are encrypted, and they will try their best to crack them. Therefore, encrypted images have a higher risk of exposure [4].

To address this issue, a possible way is to hide secret images in a carrier image so that the visual contents in the latter do not change significantly. In this way, one cracker can not perceive the existence of the secret image while the authorized users can extract the information of the secret image and restore it. This method is so-called image steganography. Image steganography is in great demand and has a wide range of applications. Currently, it is used in digital communication, copyright protection, information certification, e-commerce, and many other practical fields [5]. This can not only ensure the safe transmission of data but also provide evidence of ownership for copyright identification.

In addition, it can also identify illegal copying by adding imprints to the identities of legitimate users. Image steganography can even be applied to encrypted communications in many confidential departments involving the national economy and people’s lives, such as the military, medical care, finance, and government agencies. There are a great deal of operations for image steganography, which can be performed in both spatial and frequency domains [6,7].

However, image steganography based on spatial and frequency domains usually suffers from visual artifacts and low capacity for hiding information [8]. Since they are hand-crafted ones, it is difficult to decide which domain is used, and also difficult to find the optimal positions and strengths of hiding information [8]. In recent years, deep learning has shown its power in automatically learning useful and highly abstract features from images [9]. It also performs well in image steganography [10,11,12,13,14,15,16,17], which uses an encoding network for steganography and a decoding network for extracting secret information.

All the positions and strengths of hiding information as well as the hiding domain are automatically achieved by training the networks. However, these works have one or more of the following shortcomings: (1) the colors of the generated steganographic images are distorted [18]; (2) the hiding capacity is limited in [15,18,19,20,21]. (3) the model [15,16,17,22] does not fit the steganographic process well; and (4) the secret image is not encrypted and then steganographic in [16,21,23]; thus, it is not sufficiently secure.

A recent deep-learning model, namely Transformer, which was initially proposed for natural language processing (NLP) has also achieved promising performance in computer vision (CV) [24]. However, the potential of the Transformer in image steganography has not been investigated yet.

Motivated by the above analysis, this paper proposes a novel scheme of image steganography that uses the Transformer as hiding networks and extracting networks. Compared with previous steganography models based on Convolutional neural network (CNN), the used Transformer focuses on global information and can model longer-distance dependencies. Another advantage of the image steganography based on Transformer is that it can effectively increase the image steganography capacity. When two RGB images with the same size as the cover image are hidden, the container image can still achieve good visual effects.

In addition, a novel chaos-based image encryption algorithm that uses recursive permutation is proposed to further enhance the security of the proposed Transformers-based image steganography. The proposed Transformer and Recursive Permutation-based image Steganography is called TRPSteg. The main contributions of this paper are as follows: (1) The Transformer is introduced into image steganography, for the first time. (2) A novel chaos-based image encryption algorithm is proposed, which scrambles the pixels recursively. (3) The proposed model can realize large-capacity secret information steganography. (4) Different from common image steganography, the proposed image steganography hides an encrypted secret image instead of hiding a secret image directly. In this way, the secret image’s security is improved. (5) Extensive experiments demonstrate that the proposed image steganography significantly outperforms the state-of-the-art compared approaches.

The remainder of this paper is organized as follows: Section 2 reviews related works. We describe the proposed image steganography in detail in Section 3. The experimental results are reported and analyzed in Section 4. Finally, we conclude the paper in Section 5.

## 2. Related Works

### 2.1. Image Encryption

Due to the bulky data and high correlation of images, traditional encryption methods for common data are usually not suitable for image encryption. Since chaotic systems have the attributes of ergodicity, synchronization, and extreme sensitivity to model parameters and initial values, they have been widely applied to image encryption in recent years [25,26]. The main operations in image encryption lies in two aspects: permutation that changes the positions of pixels as well as diffusion that changes the pixels’ values [27,28]. In chaos-based image encryption, these two types of operations are determined by the generated chaotic sequences from the chaotic systems. These operations can be conducted on block-levels of pixels, pixel-level, ribonucleic acid (RNA)-level (6 bits), deoxyribonucleic acid (DNA)-level (2 bits), and bit-level data [29,30].

### 2.2. Image Steganography

There are two methods to hide information in images: watermarking and steganography; however, their goals are different. The former is usually to identify image ownership, while the latter focuses on secret communication. Two essential operations with image steganography are embedding as well as extracting. Both the operations can be performed in spatial domains and/or transform domains. LSB is such a typical spatial-domain algorithm that replaces the LSB of carrier image by the binary sequence of the secret image.

This algorithm is simple, direct, and of high embedding capacity; however, its robustness is not enough. Unlike spatial-domain algorithms, transform-domain algorithms hide secret information in the transform of images. There are many transform methods that can be applied to transform, such as discrete cosine transform (DCT), discrete Fourier transform (DFT), and discrete wavelet transform (DWT) [6]. Generally speaking, the transform-domain algorithms have better abilities to resist attacks while consuming more processing time when compared to the spatial-domain algorithms.

### 2.3. Deep Learning

As an extension of machine learning (ML), deep learning (DL) has demonstrated its advantages over traditional ML algorithms in various classification and regression tasks [31,32,33,34]. In particular, image steganography based on deep learning has begun to emerge in recent years. CNN [35] and generative adversarial network (GAN) [36] are among the most popular ones. CNN is a type of neural network that can process image data well. The idea of the CNN is to filter the uninteresting information through the convolution kernel (filter) and extract the features of the data, that is, the data we are interested in.

In [21,37], the steganography models based on CNN use an encoding network for steganography and a decoding network for extracting secret information. GANs are DL architectures typically used for generating new instances of the input data that mimic the real data. They can also be used to distinguish between real and fake data. A GAN is composed of two components: a generator network and a discriminator network. They compete against each other. The former attempts to generate fake data, while the latter focuses on identifying the reality of the fake data and improving the generator network performance. They reach the Nash equilibrium point at the end of the adversarial game [38]. Radford et al. [39] introduced deep convolutional GANs (DCGANs) in 2015.

The pioneer work of deep-learning applications in image steganography was proposed by Baluja [40], which attempted to embed a complete color image into another grayscale image of the same size, and CNNs were trained to create both hiding and extracting process and were specifically designed to work in pairs. After that, Li et al. [41] designed a more complex depth architecture for grayscale cover images and secret images to solve the distortion problem of color images. The experiments demonstrated that the method could achieve good results. Liu et al. [12] proposed a data hiding approach based on a newly proposed deep-learning model, U-Net as well as wavelet transform.

Chang et al. [42] used long short-term memory (LSTM) to realize reversible steganography model, and this neural network model significantly improved prediction accuracy and steganography distortion performance. Volkhonskiy et al. [43] used deep convolutional GAN (DCGAN) to generate image-like containers for image steganography. The most noticeable advantage of this scheme is that it can successfully deceive the steganography analyzer, and hence it can be used in real-world steganographic applications. To further solve the distortion problem, Tang et al. [44] proposed a GAN-based automatic steganographic distortion learning framework (ASDL-GAN) by using a steganographic generative subnetwork and a steganalytic discriminative subnetwork.

With this framework, the security of steganography was improved. However, ASDL-GAN still has some limitations. For example, the embedding simulator can not perfectly match the actual optimal simulator and the learning ability towards pixel-level embedding costs may not be fully used by the optimization objectives of the framework. To address these issues, Tang et al. [45] proposed another framework by combing reinforcement learning. The experimental results indicated the proposed framework could achieve state-of-the-art security performance as well as cost learning stability and efficiency.

### 2.4. Transformer

Transformer was initially proposed for the task of machine text translation by Vaswani et al. [24]. Due to its parallelization and promising performance, Transformer rapidly replaced the LSTM model and soon achieved complete dominance in NLP tasks.

The recent explosive interest in Transformer has shown that Transformer also performs well in CV. Dosovitskiy et al. [46] proposed the Vision Transformer (ViT) for image classification, which divided an image into 16×16 blocks and then stretched them into one-dimensional vectors that were fed into a network. Chen et al. [47] proposed TransUnet by combining transformer and Unet based on convolutional operations to achieve segmentation of medical images. Jiang et al. [48] used pure Transformers to build GAN, in which the architectures and training techniques were carefully designed. The proposed model achieved state-of-the-art performance on several popular datasets.

The self-attention mechanism improves the performance of many deep-learning model; however, when it is combined with Transformer, the computation complexity grows quickly, resulting in the transformer not being able to run on low computing power hardware. Liu et al. [49] proposed a new transformer model, namely Swin-Transformer, to address this issue. It uses a sliding window approach to make the network computation grow linearly and speeded up the inference of the network. In this way, the Swin-Transformer demonstrated state-of-the-art performance in many CV tasks.

### 2.5. Motivation

Image encryption and image steganography are two effective types of methods for image security. Traditional image security methods usually treat them separately. A possibly better way is to combine them to improve the security performance. Deep learning has shown its power in various CV tasks, including image steganography. Especially, as a new type of deep-learning model, Transformer and its extension, Swin-Transformer, are superior to the previous deep-learning models in NLP and CV tasks. Motivated by the super performance of Swin-Transformer, we propose an image steganography model based on Swin-Transformer. In addition, recursive permutation is proposed to further enhance image security. To the best of our knowledge, this is the first time that Transformer is applied to the task of image steganography.

## 3. TRPSteg: Transformer and Recursive Permutation-Based Image Steganography

This paper proposes a data hiding network and extraction network structure based on Transformer. To evaluate how the learned model fits the data, the loss from both the hiding network and the extraction network are weighted and summed. Before hiding the secret image into the cover image, we encrypt the secret image to prevent the leakage of the secret image information. Thus, the generated container image is double-encrypted. In order to encrypt the secret image, this paper proposes an image encryption method based on recursive permutation. After the image is encrypted, we pass the encrypted secret image and cover image to the data hiding model to generate a container image for transmission. When a receiver receives the container image, the encrypted secret image is first extracted by the extraction network, and then the encrypted image is recovered to obtain the secret image.

### 3.1. Recursive Permutation

Traditional encryption algorithms usually encrypt an entire image by treating equally some-level data, such as bit-level, two-bit-level (DNA-level), pixel-level and/or block-level data. The encryption procedure is repeated until all data have been encrypted at least once.

It is known that many repeated tasks can be solved by introducing the idea of recursion. However, few existing encryption algorithms consider using such a strategy to conduct encryption. Here, we propose a type of recursive permutation for image encryption. The operations of the recursive permutation are determined by the generated sequence $X=\{x_{0},x_{1},x_{2},\cdots \}$ of the widely used logistic chaotic system [50], defined as below:

(1) $$x_{n+1}=\lambda x_{n}\left(1-x_{n}\right),\;n=0,1,2,\cdots$$

where $x_{0}$ is an initial value in the range of [0,1] and λ is a positive parameter in the range of (0,4).

To our knowledge, it is the first time to apply recursive ideas to image encryption. The proposed encryption algorithm mainly consists of four steps, shown as follows.

Step 1. Generate a chaotic sequence the same size as the image.

Step 2. Divide the image to be encrypted into four parts: upper left, upper right, lower left, and lower right.

Step 3. Perform logistic transform encryption with the chaotic sequence on the overall image composed of four parts.

Step 4. Recursively conduct the above steps for each of the four parts until the width or height is 1.

By these four steps, a cipher image is obtained. Algorithm 1 shows the pseudocode for recursive encryption. Note that the called logistic_scramble_encryption function in Algorithm 1 refers to Algorithm 2. The decryption algorithm of recursive permutation is the inverse of the encryption algorithm.
**Algorithm 1** Recursion_encryption(img, width, height, S)**Input:** The secret image to encrypt, img; The width of image, width; The height of image, height; The generated chaotic sequence, S;**Output:** The encrypted image, img; nw ←⌊width/2⌋, nh ←⌊height/2⌋ **if** nw < 1 or nh < 1 **then**  **return** img **else**  //Divide the image into four parts, and encrypt the four parts, respectively. Encrypt the upper left part.  **Recursion_encryption**(img[0:nw, 0:nh, :], nw, nh, S)  //Use the logistic_scramble_encryption function (Algorithm 2) to scramble the image with the chaotic sequence generated by the logistic algorithm.  img[0:nw, 0:nh] ← **logistic_scramble_encryption**(img[0:nw, 0:nh], S)  //Encrypt the lower left part.  **Recursion_encryption**(img[0:nw, nh:height], nw, nh, S)  img[0:nw, nh: height] ← **logistic_scramble_encryption**(img[0:nw, nh: height], S)  //Encrypt the upper right part.  **Recursion_encryption**(img[nw:width, 0:nh], nw, nh, S)  img[nw:width, 0:nh] ← **logistic_scramble_encryption**(img[nw:width, 0:nh], S)  //Encrypt the lower right part.  **Recursion_encryption**(img[nw:width, nh:height], nw, nh, S)  img[nw:width, nh:height] ← **logistic_scramble_encryption**(img[nw:width,nh:height], S)  //Encrypt the entire image.  img[0:width, 0:height] ← **logistic_scramble_encryption**(img[0:width, 0:height], S) **end if** **return** img

**Algorithm 2** logistic _scramble_encryption(img, S)**Input:** The image to encrypt, img; The generated chaotic sequence, S;**Output:** The encrypted image, img; w, h ← img.shape //Get the width (w) and height (h) of img. img ← img.flatten() //Convert img to 1D array. idx ← sort(S) //Sort S to obtain the corresponding indices (idx) of S. img ← img[idx,:] img ← img.reshape(w,h,3) **return** img

### 3.2. Hiding Network

**Figure 1:**
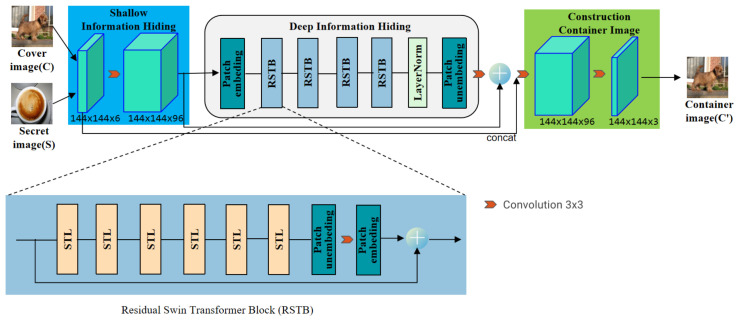
The architecture of the hiding network.

The hiding network uses a neural network structure based on the Swin-Transformer to hide the secret image into the cover image. The specific structure is shown in Figure 1. An RGB cover image and an RGB secret image are used as network input and an RGB container image is used as the network output. All these three images have the same size of 144×144×3. The hiding network consists of three modules: shallow information hiding, deep information hiding, and construction container image modules. Shallow information hiding module uses a 3×3 convolution layer. The convolution layer is good at early visual processing, leading to more stable optimization and better results [51].

This also provides a simple way to map the input image space to a high-dimensional feature space. Then, the deep information hiding module composed of one Patch Embedding, four residual Swin-Transformer blocks (RSTB), one LayerNormal, one Patch Unembedding, and a 3×3 convolution layer, which is used to hide deep information of the images. Finally, the construction container image module uses a 3×3 convolutional layer to construct the container image with the size of 144×144×3.

**Figure 2:**
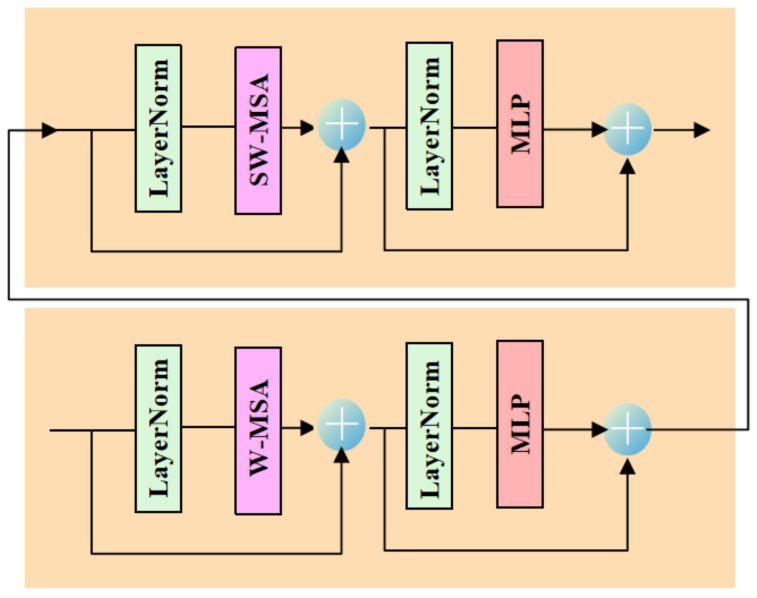
Two successive Swin-Transformer Layers (STL).

**Figure 3:**
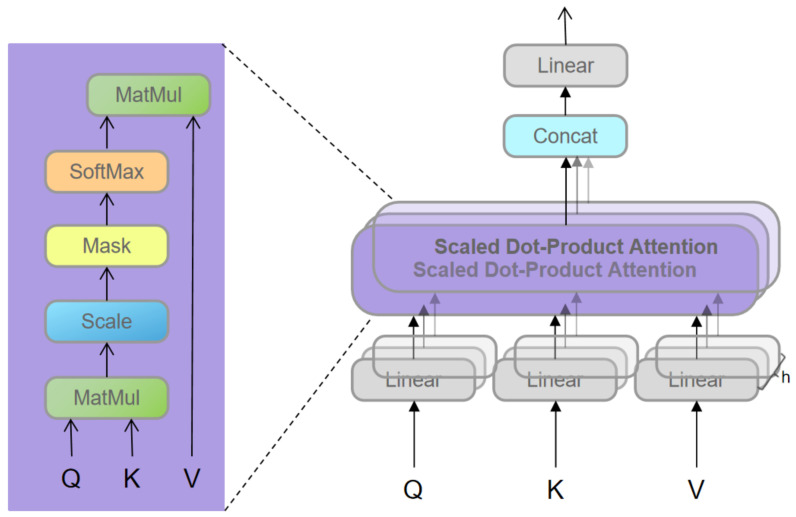
Self-attention calculation process.

As shown in Figure 1, RSTB is a residual block with Patch unembedding, Patch embedding, Swin-Transformer layer (STL) and convolutional layer. STL is based on the standard multi-head self-attention of the original Transformer layer [24,49]. The main differences lie in local attention and the shifted window mechanism. As shown in Figure 2, given an input image of size H×W×C, Swin-Transformer first reshapes the input to a $\frac{HW}{M^{2}}\times M^{2}\times C$ feature by partitioning the input into non-overlapping M×M local windows, where $\frac{HW}{M^{2}}$ is the total number of windows. Then, calculate the standard self-attention for each window, i.e., local attention. For a local window feature $X\in R^{M^{2}\times C}$, the query, key and value matrices *Q*, *K* and *V* are computed as:

(2) $$\begin{array}{c} Q=XP_{Q},K=XP_{K},V=XP_{V}, \end{array}$$

where $P_{Q}$, $P_{K}$ and $P_{V}$ are projection matrices that are shared across different windows. Generally, we have $Q,K,V\in R^{M^{2}\times d}$. As shown in Figure 3, the attention matrix is thus computed by the self-attention in a local window as

(3) $$\begin{array}{c} Attention\left(Q,K,V\right)=SoftMax\left(\frac{QK^{T}}{\sqrt{d}}+E\right)V, \end{array}$$

where *E* is the learnable relative positional encoding. In practice, following [24], we perform the attention function six times in parallel and concatenate the results for multi-head self-attention (MSA).

Next, a multi-layer perceptron (MLP ) that has two fully connected layers with GELU non-linearity between them is used for further feature transformations. The LayerNorm (LN) layer is added before both MSA and MLP, and the residual connection is employed for both modules. The whole process is formulated as:

(4) $$\begin{array}{c} X=MSA(LN(X))+X, \end{array}$$

(5) $$\begin{array}{c} X=MLP(LN(X))+X. \end{array}$$

**Figure 4:**
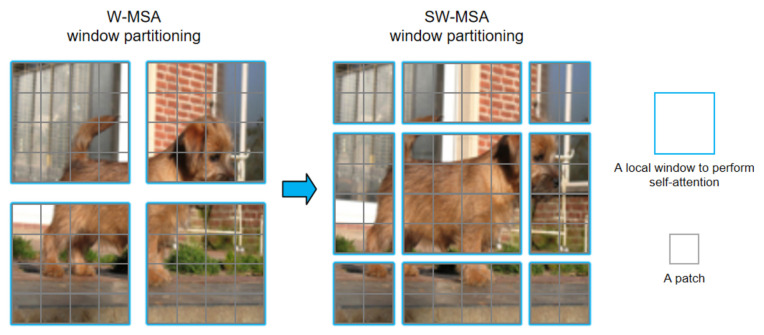
Approach for computing self-attention in the proposed Swin-Transformer architecture.

However, when the partition is fixed for different layers, there is no connection across local windows. Therefore, regular and shifted window partitioning are used alternately to enable cross-window connections [49], where shifted window partitioning means shifting the feature by pixels before partitioning. In order to enable cross-window, the number of STL modules must be even. Figure 2 shows the two successive Swin-Transformer blocks. From Figure 4, in W-MSA window partitioning, a regular window partitioning scheme is adopted, and self-attention is computed within each window. In SW-MSA window partitioning, the window partitioning is shifted, resulting in new windows.

The self-attention computation in the new windows crosses the boundaries of the previous windows in W-MSA window partitioning, providing connections among them. In two successive Swin-Transformer layer, the first module uses a regular window partitioning strategy which starts from the top-left pixel, and the 8×8 feature map is evenly partitioned into 2×2 windows of size 4×4(M=4). Then, the next module adopts a windowing configuration that is shifted from that of the preceding layer by displacing the windows by $\left(\left\lfloor \frac{M}{2},\frac{M}{2}\right\rfloor \right)$ pixels from the regularly partitioned windows. W-MSA and SW-MSA denote window based multi-head self-attention using regular and shifted window partitioning configurations, respectively.

### 3.3. Extraction Network

**Figure 5:**
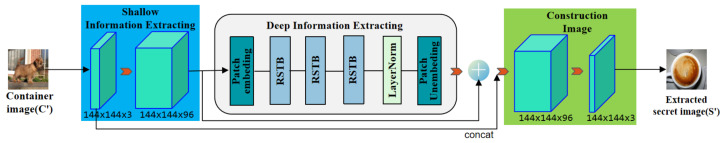
The architecture of the extracting network.

The extracting network is similar to the hiding network, which also uses a neural network based on Swin-Transformer structure to extract the secret image. The similar network structure can promote the image decryption performance. The specific structure is shown in Figure 5. An RGB container image with a size of 144×144×3 is used as network input and an RGB extracted secret image with a size of 144×144×3 is used as the output. The difference between the extraction network and the hiding network is that the latter uses three residual Swin-Transformer blocks in order to speed up image decryption while maintaining good image decryption performance.

### 3.4. Loss Function

The evaluation criteria of traditional image data hiding schemes include peak signal-to-noise ratio (PSNR), mean squared error (MSE), etc., which are used to quantify the difference between the original cover image and the container image, and the difference between the secret data and the extracted data. Therefore, the MSE is used as the model loss function in this paper. In the hiding network, MSE is used to measure the difference between the cover image *C* and the container image $C^{\prime }$, while in the extracting network, the MSE is used to measure the difference between the secret image *S* and the extracted secret image S′. The MSE function equation can be formulated below:

(6) $$\begin{array}{c} MSE\left(I,I^{\prime }\right)=\frac{1}{M\times N}\sum_{i=1}^{M}\sum_{j=1}^{N}{\left(I_{i,j}-I_{i,j}^{\prime }\right)}^{2}, \end{array}$$

where *I* and $I^{{}^{\prime }}$ denote two matrices for MSE operation, and *M* and *N* denote the length and width of the matrix, respectively. The loss function of the data hiding network is defined as:

(7) $$\begin{array}{c} Loss=MSE\left(C,C^{\prime }\right)+\beta \times MSE\left(S,S^{\prime }\right), \end{array}$$

where $MSE(C,C^{{}^{\prime }})$ and $MSE(S,S^{{}^{\prime }})$ are the cost of the hiding network and the extraction network, respectively, β is a tradeoff factor to balance these two types of loss. Here, the weight of the error term $MSE(C,C^{{}^{\prime }})$ of the hiding network is not shared with the weight of the extraction network, and the weight of the error term $MSE(S,S^{{}^{\prime }})$ is shared between the two networks. This ensures that the two networks adjust the network training by receiving this error term to minimize the error loss of the hiding network reconstructed secret image and the cover image, and to ensure that the information of the secret image is completely encoded on the cover image.

### 3.5. Flowchart

**Figure 6:**
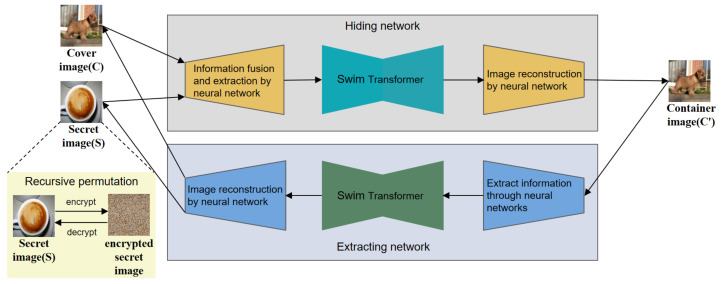
The overall architecture diagram of the proposed TRPSteg scheme.

Figure 6 shows the overall architecture diagram of the proposed TRPSteg. TRPSteg consists of four modules: Hiding network and Extarcting network based on Swim-Transformer, Encryption and Decryption based on recursive permutation. When an image needs to be encrypted and transmitted, in order to prevent the leakage of the image information, the image is steganographically stored in a natural image. The secret image can be directly hidden in the cover image, or it can be encrypted using the proposed recursive encryption algorithm and then passed into the Hiding network model. The Hiding network uses the Swim-Transformer and CNN to fuse the cover image and secret image into the container image, the visual effect of the container image and cover image is almost the same, and the container image contains the information of the secret image.

We use the container image generated by the Hiding network to transmit the secret image information to achieve the effect of steganographic encryption. When the receiver receives the container image, it can decrypt the container image through the Extracting network to obtain the secret image. To extract the original image better, the Extracting network adopts a model architecture based on Swim-Transformer and CNN similar to the Hiding network. Before passing the secret image into the Hiding network, the secret image can be encrypted by recursive permutation algorithm to prevent the leakage of the secret image information when the model is attacked and to prevent the loss of key information caused by the loss of local information during transmission.

### 3.6. Differences between TRPSteg and Other Image Steganography Schemes

The proposed TRPSteg uses a type of deep-learning model, Transformer, for image steganography, and thus it is different from traditional methods with spatial and frequency domains. The used Swim-Transformer focuses on global information and can model longer-distance dependencies, while CNN focuses on local information and has a weak ability to capture global information. At the same time, in previous scheme [15,16,17], the extracting network is generally the most basic CNN, and the encryption process and the decryption process are difficult to match. The proposed scheme replaces the extraction network with a structure similar to the hiding network, which improves the performance of the decryption network. Therefore, the proposed TRPSteg is also clearly different from the previous CNN-based image steganography schemes [15,16,17]. In addition, a novel strategy of recursive permutation is proposed to encrypt the secret image and further improve the security of the steganography model.

## 4. Experiments

### 4.1. Experimental Setting

In this work, 45,000, 5000, and 5000 images from the ImageNet [52] are used for model training, validating and testing, respectively. The results of all the following indicators are performed on the testing set.

The Adam optimization method is used to automatically adjust the learning rate so that the network parameters can be learned smoothly. The experimental environment is python3.6+pytorch, and the hardware uses GPU: NVIDIA GeForce 2080 Ti. In the training process, the following optimal parameters are obtained: the initial network learning rate lr=0.0001, the task weight β=1, and the number of iterations epoch 200. The initial value of $x_{0}$ and μ of the logistic map are set to 0.51 and 3.7, respectively. These parameters can also be optimized by various evolutionary algorithms [53]. The source code of the proposed TRPSteg is available at https://github.com/Zmingcheng/Swim-image-steganography (accessed on 23 June 2022).

### 4.2. Visual Effect

**Figure 7:**
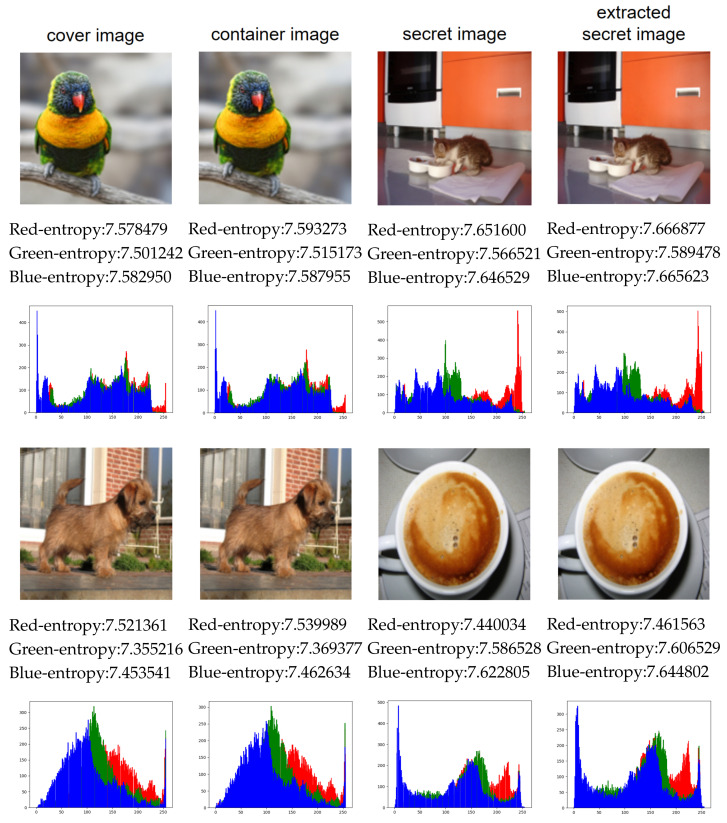
Comparison of experimental images in three aspects: visual effect, three-channel information entropy, and histogram.

Figure 7 shows the experimental images and the corresponding pixel histograms, including the cover image, container image, secret image, and extracted image. All images are color images with a size of 144×144. According to Figure 7, it can be seen that the visual difference between the cover image and the container image is not obvious, and almost no visual difference between the secret image and the extracted image can be seen. In addition, we report the entropy of each channel of each image in the figure. It can be seen that there is little difference between cover image and container image, secret image and extracted secret image in the information entropy value of the three channels. The small difference in information entropy indicates that the amount of information contained in the two images is almost the same.

In order to show the distribution of pixel values and the degree of modification of all images, we analyze the pixel histograms. According to the histograms in Figure 7, there is no clear difference between the cover image and the container image in pixel histogram. At the same time, we cannot see a clear difference between the secret image and the extracted image by the proposed scheme. Therefore, the proposed scheme can achieve good performance in visual effect.

### 4.3. Security Analysis

Generally speaking, the residual image can directly show the visual difference between the cover image and container image, and it can be used to analyze whether the container image contains semantic information about the secret image. The scheme will be said to be insecure once the residual image contains semantic information about the secret image.

**Figure 8:**
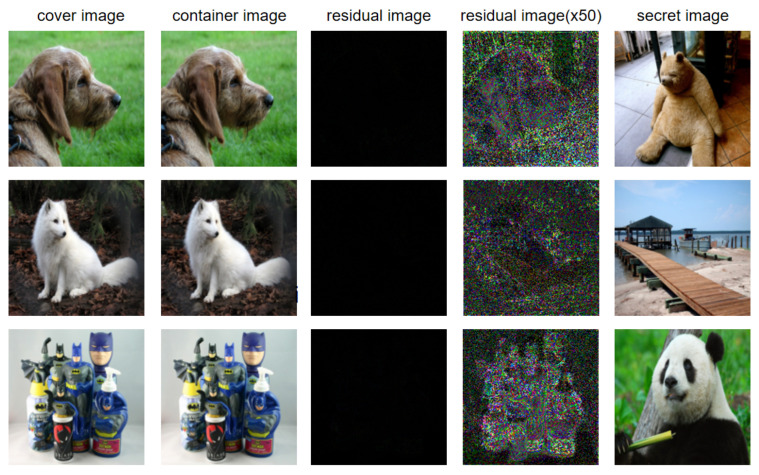
The residual image between the cover image and the container image, and the secret image.

Figure 8 shows the cover image, container image, residual image between the cover image, and the container image, the residual image enlarged by 50 times, and the secret image. We can easily find that the residual image does not have any visual information, even when the residual image is magnified by a factor of 50. Thus, it is difficult to obtain the useful semantic information from the residual image with this scheme, and the leakage of secret data due to residual images could be avoided. Compared with the method proposed in [40], the proposed steganography method improves the security greatly.

According to the histogram comparison in Figure 7, the histogram of the hidden image have no correlations with the histogram of the secret image. It is difficult to judge whether the secret data is hidden in the image, and the secret image can not be extracted according to the histogram. The container image clearly indicates good visual quality and offers no clues to the presence of any hidden information even with statistical analysis. Therefore, the security of the proposed steganography scheme is relatively high.

### 4.4. Image Quality

There exist many image quality assessment metrics, such as PSNR, structural similarity (SSIM) [54,55], feature similarity (FSIM) [56], and gradient magnitude similarity deviation (GMSD) [57]. However, PSNR and SSIM are two most widely used evaluation metrics for image steganography. Following the previous image steganography, this paper also uses these two metrics to evaluate the quality of the generated images.

PSNR is an objective evaluation index of image quality, which is widely used in data hiding. A higher PSNR value indicates that the image distortion is small, and the image quality after hiding is better. It is one of the most essential parameters to judge the effectiveness of any steganography scheme. PSNR is mainly used to measure the distortion rate of an image and display it as a score. Its definition is based on the MSE and can be formulated as below:

(8) $$\begin{array}{c} PSNR=10\times \log_{10}\left(\frac{{\left(2^{n}-1\right)}^{2}}{MSE(I,I_{a})}\right), \end{array}$$

where *I* is the cover image or the original secret image and $I_{a}$ is the container image or the extracted secret image, accordingly. The calculation process of MSE is shown in Equation (6).

SSIM index is a metric based on the human visual system (HVS) to quantify the degradation of structural information between two images. It evaluates the processed image quality by comparing the brightness, contrast, and structural similarity of the original image. SSIM can be formulated as below:

(9) $$\begin{array}{c} SSIM\left(x,y\right)=\frac{\left(2\mu_{x}\mu_{y}+c_{1}\right)\left(2\sigma_{xy}+c_{2}\right)}{\left(\mu_{x}^{2}+\mu_{y}^{2}+c_{1}\right)\left(\sigma_{x}^{2}+\sigma_{y}^{2}+c_{2}\right)}, \end{array}$$

where *x* represents a cover image or secret image, *y* represents the container image or the extracted secret image, $\mu_{x}$ and $\mu_{y}$ represent the pixel average, $\sigma_{x}^{2}$ and $\sigma_{y}^{2}$ represent the variance of pixel values, $\sigma_{xy}$ is determined by the correlation between the image blocks *x* and *y*, $c_{1}={\left(k_{1}L\right)}^{2}$, $c_{2}={\left(k_{2}L\right)}^{2}$ is a constant used to maintain stability, and *L* is the range of pixel values. $k_{1}$ and $k_{2}$ are usually set to 0.01 and 0.03 by default, respectively.

To better evaluate the image quality, we divide the proposed image steganography into three types: TRPSteg_H1, TRPSteg_H2 and TRPSteg_Enc denote hiding one secret image, two secret images, and an encrypted secret image with recursive permutation, respectively.

Table 1 shows the average PSNR and SSIM of the proposed models on the testing set, and they are also compared with some latest neural network data hiding schemes. The lower PSNR value, the more serious image distortion. For PSNR values lower than 30 dB, it is generally considered that the visual effect of the image is poor. The value of SSIM is between −1 and 1. As the SSIM value decreases, the involved two images become increasingly irrelevant.

From Table 1, it can be found that the PSNR values of the proposed schemes are all greater than 30 dB; thus, the container image and the extracted image have good visual effects. Compared with other schemes, TRPSteg_H1 can achieve the highest PSNR and SSIM values when hiding an image. The PSNR value is more than 45 dB, and the value SSIM is more than 0.99. Even if two images are hidden, TRPSteg_H2 also can achieve high PSNR and SSIM values, even higher than some schemes that hide one image.

Both [41] and TRPSteg_Enc encrypt the secret image and then pass it into the model. The secret image proposed in [41] is a grayscale image, while that of TRPSteg_Enc is a color image. In TRPSteg_Enc, the PSNR of the cover image can be higher than 40 dB, and the PSNR and SSIM values of the secret image are even higher than the scheme proposed in [41].

### 4.5. Hidden Capacity Analysis

The data embedding capacity, termed as *EC*, basically measures the strength or capability of how many bits can be concealed within a single pixel of a cover image. *EC* is the most important parameter that ensures the quality of a steganography technique, which can be defined as below:

(10) $$\begin{array}{c} EC=\frac{NS}{NC}, \end{array}$$

where NS represents the number of concealed bits, while NC represents the number of pixels in the cover image.

Table 2 shows the *EC* comparison between the proposed scheme and some other schemes, including traditional schemes and neural network schemes. According to this table, we find that neural network steganography methods have a larger effective capacity than traditional data hiding schemes [19,20,59]. The maximum capacities of the traditional data hiding schemes [19,20,59] and the neural network steganography methods [15,18,21] are 2 and 24 bpp.

The proposed scheme not only achieves the maximum ability of the schemes [15,18,21] but also makes the *EC* value reach 48 bpp. When hiding two images, the container still maintained a good visual performance. We calculate the average PSNR and SSIM values for the container and extracted secret images. As shown in Table 1, the average PSNR and SSIM values of the cover and secret images decreases as the number of hidden images increases. Clearly, with the increase of hidden images, it will be more difficult to hide hidden images into a cover image.

Nevertheless, when hiding two images into an cover image, the proposed TRPSteg can also achieve high PSNR and SSIM values and the container images are with good visual imperceptibility.

### 4.6. Parameter Influence and Ablation Study

In this subsection, we mainly discuss various factors that affect the training results, including the setting of the parameter β of the loss function in (7), the selection of the extracting network model, the number of RSTB modules and the number of STL in RSTB.

Table 3 shows the experimental results of the proposed scheme with different parameter β of the loss function. By simply adjusting the parameter β of the loss function, our model can obtain a more ideal container image and an extracted secret image. When the parameter β is adjusted from 0.75 to 1, the PSNR and SSIM values of the container image and extracted secret image were improved, and the PSNR value of extracted image was improved by 1.5 dB. When the value of β continues to increase, the performance of the model is not improved any more. Hence, the proposed scheme sets the parameter β of the loss function to 1.

Table 4 shows the experimental results of different extracting network models, different numbers of RSTB modules and different numbers of STL modules in RSTB. From this table, it can be seen that as the network depth and complexity decrease, the effect of the image steganography scheme also decreases. Compared with the previously ordinary convolutional neural extraction network, the proposed scheme uses Swim-Transformer for the extracting network. The extraction effects are significantly improved, and the hiding network has similar effects.

### 4.7. Statistical Test

**Figure 9:**
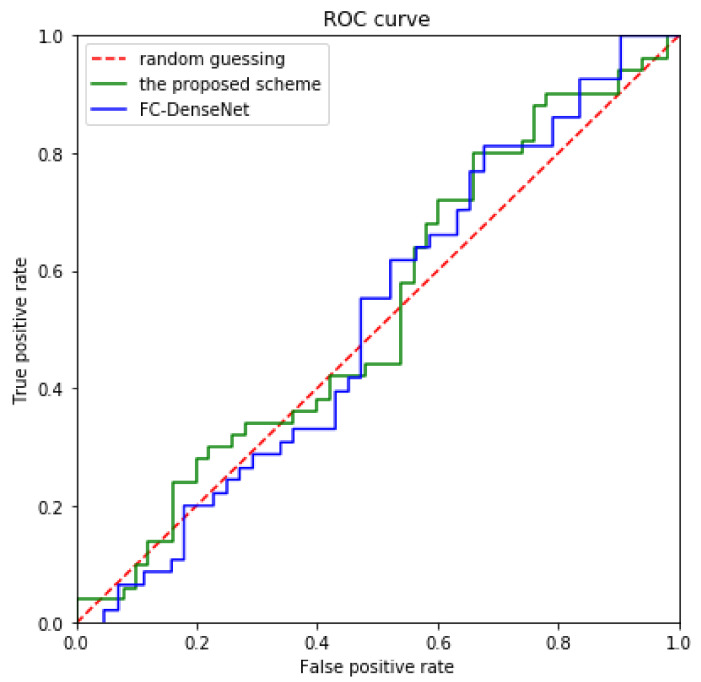
Comparison of the ROC curves drawn by the proposed scheme and FC-DenseNet [16] using the StegExpose analysis tool.

StegExpose combines multiple statistical indicators, such as Chi-Square and regular singular (RS) analysis, which plays a crucial role in image steganalysis [60]. We use StegExpose with a standard threshold of 0.2 to analyze the proposed scheme, and the results are shown in Figure 9. The horizontal axis indicates that an image that is not contain secret information is judged as a steganographic image, and the vertical axis indicates that an image that contain secret information is judged as a steganographic image. The red dashed line represents random guessing.

The blue and green solid line represent the receiver operating characteristic (ROC) curves drawn by FC-DenseNet [16] and the proposed scheme, respectively. By observing Figure 9, it can be found that the ROC curve of the proposed scheme are similar to FC-DenseNet [16], and even our green solid line is closer to the red dotted line; therefore, the analysis of the proposed scheme using the StegExpose is only slightly better than random guessing, which shows that the proposed scheme can effectively prevent the analysis of this steganographic tool.

### 4.8. Threats to Validity

Image steganography is to hide secret images in a cover image, however, maintaining the containing image that carries the secret image information as visually identical to the cover image. Since the maximum amount of information that an image can hold is limited, the amount of secret information that a containing image can contain is also limited. The possible threats to the proposed TRPSteg can be generally classified as internal validity and construct validity [61].

Internal validity reflects how changes in one factor can lead to changes in another related factor. In our experiments, the two main evaluation metrics for steganographic quality are hidden capacity and image quality. These two factors restrict each other, and changes in any one factor will lead to changes in the other. When we increase the capacity of the information, there is no drastic changes in the quality of the image. However, when comparing PSNR and SSIM values, Table 1 shows that the changes in image quality are small.

Construct validity is to validate the measurements. In our experiments, histogram graphs, PSNR and SSIM values are used to accurately measure the changes in the quality of the image. These values are rechecked for correctness. Comparing the PSNR and SSIM values achieved by the proposed TRPSteg with some state-of-the-art steganography models, Table 1 shows that the proposed image steganography significantly outperforms the others.

### 4.9. Discussion

In order to solve the problem of secret information leakage, the secret image to be hidden is first, encrypted by the proposed recursive permutation, and then the cover image and encrypted secret image are integrated as a container image. Since the semantic content of the secret image is scrambled before they are embedded into cover images, the confidentiality of secret information is well protected.

**Figure 10:**
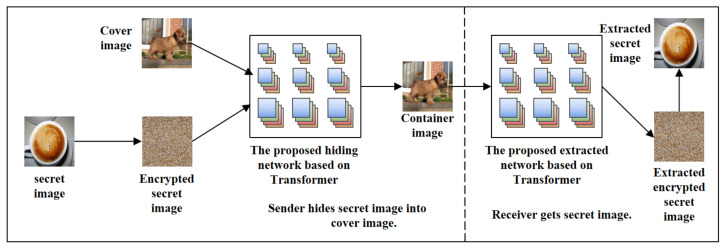
The recursive permutation encryption steganography model architecture.

**Figure 11:**
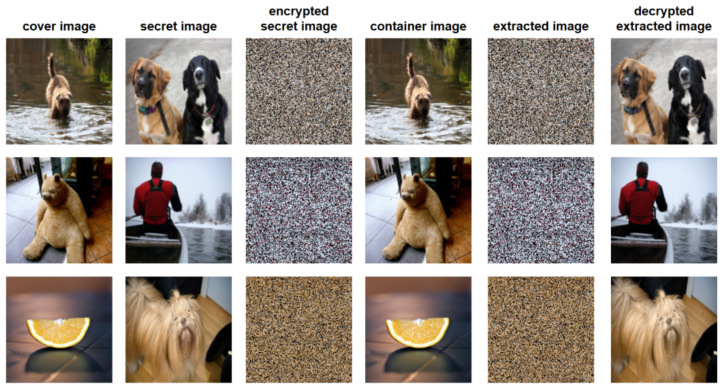
The experimental results obtained from randomly selected images from the ImageNet dataset by the recursive permutation encryption steganography scheme.

Figure 10 shows the architecture diagram of the model. We use the ImageNet dataset to train and test the model. The PSNR and SSIM are used to evaluate image quality. Table 1 lists the PSNR values and SSIM values of this scheme. From this table, we can find that even if the secret image is recursively encrypted and then passed into the steganography model, good results can still be obtained. The value of PSNR values are higher than 40 dB, and the values of SSIM are higher than 39 dB. Some examples are shown in Figure 11. Note that the third and fifth columns are the encrypted secret image and extracted image, respectively. Applying the decrypted operations of the proposed recursive permutation, we can finally obtain the decrypted extracted image, as shown in the sixth column.

**Figure 12:**
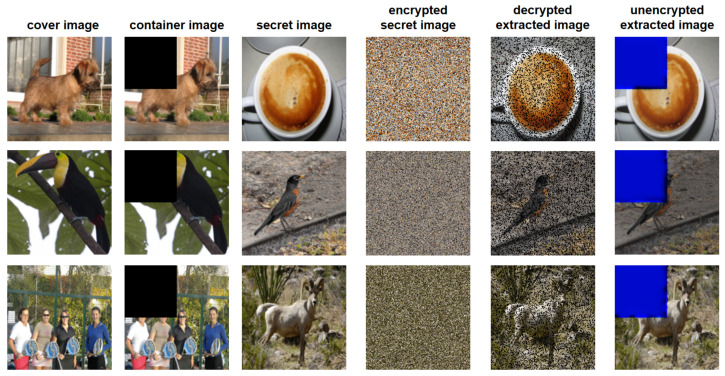
Effect picture of the information loss comparison experiment.

To demonstrate the robustness of the proposed image steganography scheme, we crop the $\frac{1}{4}$ data of the container images at the upper left corner. The corresponding images are shown in Figure 12, and the decrypted extracted images are shown in the fifth column of Figure 12. From this figure, we can see that, even for a large percentage of data loss, the proposed scheme can still recover the secret images with visually meaningful information. It indicates that the proposed scheme can resist attacks of data loss.

## 5. Conclusions

Image steganography has shown its advantages over secure communication. As a recent deep-learning model, the Transformer demonstrated its superiority to computer vision tasks. This paper proposes a novel image steganography scheme based on the Swim-Transformer, with which novel embedding networks and extraction networks are designed. In addition, a recursive permutation is proposed to scramble the secret image to further enhance the security. The experiments indicate that the Transformer outperformed the compared models in terms of the evaluation indicators. The secret image can be encrypted before embedding, and there was no significant difference in the visual effects of the carrier image and the extracted image, showing that the proposed image steganography with encrypted images embedded is feasible.

This work is a new attempt to simultaneously use Transformers and encryption techniques for image steganography. The extensive experiments have demonstrated the effectiveness of the Transformer network model in the field of image steganography. It also significantly outperforms the state-of-the-art compared approaches. The performance of the steganography model can be effectively improved by building an extraction network with a similar structure to the hiding network. In addition, the proposed scheme combines chaotic image encryption with the Transformer-based image steganography, which further improves the security of the scheme. At the same time, the proposed recursive permutation strategy can be widely used in image encryption. All these attributes make the proposed image steganography have good applicability.

In the future, we will study how to improve the quality of the container images and compress hiding and extracting model sizes. In addition, we will add the SSIM value to error metrics for training the networks to make the error metrics more closely associated with human vision. We will also study merging several image quality assessment metrics into one to evaluate image steganography schemes.

## Figures and Tables

**Table 1:** Average values of SSIM and PSNR of different steganography schemes.

Schemes	*EC* (bpp)		Cover Image	Secret Image
Rehman et al. [18]	24	PSNR	32.5	34.7571
		SSIM	0.9371	0.93
Li et al. [41]	8	PSNR	42.3	38.45
		SSIM	0.987	0.953
Duan et al. [23]	24	PSNR	40.4716	40.6665
		SSIM	0.9794	0.9842
Liu et al. [12]	8	PSNR	39.7708	43.3571
		SSIM	0.9828	0.9862
Baluja et al. [40]	24	PSNR	41.2	37.6
		SSIM	0.98	0.97
Lu et al. [58]	24	PSNR	38.05	35.38
		SSIM	0.954	0.955
Nao et al. [16]	24	PSNR	39.556	37.092
		SSIM	0.985	0.975
Duan et al. [22]	24	PSNR	40.211	37.04
		SSIM	0.993	0.983
Gan et al. [17]	8	PSNR	38.74	37.9
		SSIM	0.968	0.9713
Zeng et al. [15]	24	PSNR	43.57	38.14
		SSIM	0.987	0.967
TRPSteg_H1	24	PSNR	45.1918	44.568
		SSIM	0.9918	0.9936
TRPSteg_H2	48	PSNR	40.7474	36.6029
		SSIM	0.9809	0.9694
TRPSteg_Enc	24	PSNR	40.2816	38.5234
		SSIM	0.9795	0.9718

**Table 2:** Steganographic capacity comparison.

	Schemes	*NC*	*NS*	*EC*
Traditional	Gao et al. [59]	256×256	132×126	2
	Meng et al. [19]	512×512	256×256×8	2
	Pakdaman et al. [20]	512×512	128×128×8	0.5
Neural network	Rehman et al. [18]	300×300(RGB)	300×300×8	8
	Zhang et al. [21]	256×256(RGB)	256×256×8	8
	Zeng et al. [15]	256×256(RGB)	256×256×3×8	24
	TRPSteg_H1	144×144(RGB)	144×144×3×8	24
	TRPSteg_H2	144×144(RGB)	2×144×144×3×8	48
	TRPSteg_Enc	144×144(RGB)	144×144×3×8	24

**Table 3:** The SSIM and PSNR values of difference parameter β of the loss function.

β	Container Image	Extracted Image
0.75	44.88/0.991	43.05/0.991
1.00	45.19/0.992	44.57/0.994
1.25	45.18/0.991	44.57/0.994

**Table 4:** The SSIM and PSNR values of different modules.

Hiding Network	Extraction Network	Container Image	Extracted Secret Image
4,4,4,4 (RSTB)	4,4,4,4 (RSTB)	42.25/0.985	41.78/0.989
6,6 (RSTB)	6,6 (RSTB)	39.50/0.974	38.73/0.981
6,6,6,6 (RSTB)	CNN	43.10/0.988	39.95/0.986
TRPSteg_H1	TRPSteg_H1	45.19/0.992	44.57/0.994

## Data Availability

The used test images are all included in the paper.
